# Working Memory Deficits in Hippocampal Amnesia are Associated with Apathy

**DOI:** 10.1523/JNEUROSCI.0543-26.2026

**Published:** 2026-07-20

**Authors:** Bahaaeddin Attaallah, Maria Raquel Maio, Younes A. Tabi, Lisa Nobis, Rhea Zambellas, Sofia Toniolo, Sarosh R. Irani, Sanjay G. Manohar, Masud Husain

**Affiliations:** ^1^ Department of Brain Sciences, Imperial College London, London W12 0NN, United Kingdom; ^2^Nuffield Department of Clinical Neurosciences, University of Oxford, Oxford OX3 9DU, United Kingdom; ^3^Department of Experimental Psychology, University of Oxford, Oxford OX1 3PH, United Kingdom; ^4^Departments of Neurology and Neurosciences, Mayo Clinic, Jacksonville, Florida 32224

**Keywords:** apathy, autoimmune encephalitis, functional connectivity, hippocampus, memory misbinding, working memory

## Abstract

The hippocampus is increasingly recognized for its role in working memory representations and goal-directed behavior, yet whether these functions share common neural substrates remains unclear. We investigated short-term memory performance in 27 patients (21 males and 6 females) with LGI1-antibody limbic encephalitis, a condition with predominant hippocampal involvement, compared to 27 age- and gender-matched healthy controls, using an object–location continuous report task with Bayesian mixture-modeling. Patients exhibited predominantly elevated misbinding errors—incorrectly associating objects with non-target locations—with unaltered guessing and only marginally reduced precision. Critically, apathy was positively associated with misbinding exclusively in patients, independent of depression and global cognition. Neuroimaging in a subset of patients (*n* = 12) revealed reduced hippocampal volumes and preliminary evidence for diminished hippocampal—medial prefrontal connectivity, which was associated with both higher apathy and increased misbinding at short retention intervals. These findings demonstrate that hippocampal dysfunction produces convergent deficits in memory binding and motivation, potentially reflecting disruption of a common brain mechanism linking working memory processing to goal-directed behavior.

## Significance Statement

The hippocampus, a brain region long associated with memory, is increasingly recognized for its role in goal-directed behavior. We tested patients with autoimmune limbic encephalitis, a condition that primarily damages the hippocampus, on a working memory task that distinguishes different types of errors. Patients showed a predominant impairment in binding object identities to their locations, and these binding errors correlated with apathy—loss of motivation—independent of depression and general cognition. Reduced functional connectivity between the hippocampus and prefrontal cortex was associated with both higher apathy and greater binding errors. Our findings suggest that the hippocampus supports both memory and motivation through common neural mechanisms, with potential clinical implications for cognitive and motivational symptoms in hippocampal disorders.

## Introduction

The hippocampus has long been considered essential for long-term memory, with working memory traditionally attributed to prefrontal and parietal cortical systems (Baddeley, 2003; Squire et al., 2004). However, accumulating evidence challenges this strict dichotomy, demonstrating critical hippocampal contributions to working memory when tasks require the binding of features into integrated representations (Hannula et al., 2006; Olson et al., 2006; Yonelinas, 2013). This binding function, which links separate elements such as objects and their locations into coherent memory traces, appears to involve hippocampal computations regardless of retention interval, suggesting a fundamental role in short-term processing (Cowan, 2001; Ranganath and Blumenfeld, 2005; Husain, 2024).

Patients with hippocampal damage show preserved memory for individual features but impaired memory for feature conjunctions, even at short delays (Olson et al., 2006; Pertzov et al., 2013). Computational models applied to continuous report tasks have enabled precise quantification of these failures, distinguishing misbinding errors from random guessing and degraded precision (Bays and Husain, 2008; Bays et al., 2009; Toniolo et al., 2025b). Elevated misbinding rates have been observed following hippocampal lesions (Pertzov et al., 2013), in healthy ageing (Pertzov et al., 2015), and in Alzheimer’s disease (AD; Zokaei et al., 2020; Toniolo et al., 2025a,b), establishing misbinding as a sensitive marker of hippocampal-dependent working memory processing.

Beyond its role in memory, the hippocampus is increasingly recognized for its contribution to goal-directed behavior and decision-making (Tracy et al., 2001; Gilboa et al., 2018; Bakkour et al., 2019; Attaallah et al., 2022, 2024b). The hippocampus maintains dense reciprocal connections with prefrontal cortex, anterior cingulate, and ventral striatum—regions central to reward processing and motivation (Floresco et al., 1997; Thierry et al., 2000). Hippocampal activity is modulated by reward anticipation and motivational state (Adcock et al., 2006; Shohamy and Adcock, 2010), and hippocampal–prefrontal connectivity supports the integration of mnemonic and motivational information during adaptive behavior (Preston and Eichenbaum, 2013; Eichenbaum, 2017). These observations suggest that hippocampal dysfunction could simultaneously impair memory binding and disrupt motivational processes through shared neural mechanisms.

Apathy is a motivational deficit of goal-directed behavior (Husain and Roiser, 2018; Le Heron et al., 2019; Lanctot and Aleman, 2021). Whilst traditionally attributed to frontal-subcortical circuit dysfunction (Levy and Dubois, 2006), apathy is prevalent in conditions affecting the hippocampus, including AD (Nobis and Husain, 2018; Lanctot and Aleman, 2021; Attaallah et al., 2024a) and autoimmune encephalitis (van Sonderen et al., 2016). Recent frameworks propose that apathy reflects impaired prospective cognition and inferential planning (Eggins et al., 2022; Williams and Rowe, 2025), processes that critically involve the hippocampus (Buckner, 2010; Schacter et al., 2017). Thus, if the hippocampus supports both the binding of features in working memory and aspects of prospective inference (e.g., simulation of possible futures) that motivate behavior, then hippocampal pathology might produce correlated deficits in short-term memory and motivation.

LGI1-antibody limbic encephalitis (aLE) provides a unique opportunity to test this hypothesis. This condition is characterized by antibody-mediated inflammation predominantly affecting the hippocampus and adjacent temporal structures (Irani et al., 2010; Graus et al., 2016; van Sonderen et al., 2016), resulting in highly focal hippocampal atrophy in the chronic stages (Miller et al., 2017; Spanò et al., 2020a,b). Patients frequently exhibit persistent memory impairment and neuropsychiatric symptoms, including apathy (van Sonderen et al., 2016; Finke et al., 2017; Binks et al., 2021; Kelly et al., 2025). Reduced hippocampal–prefrontal connectivity has been associated with memory impairment across conditions (Wang et al., 2006; Salami et al., 2014) and with apathy in Parkinson’s disease, stroke, and AD (Skidmore et al., 2013; Attaallah et al., 2024a). Whether similar disruptions in aLE contribute to both binding deficits and apathy remains unknown.

In this study, we investigated short-term memory binding in patients with aLE and matched healthy controls (HC) using an object–location continuous report task. Performance was analyzed with Bayesian mixture-modeling to dissociate misbinding errors, guessing, and memory precision. We then examined associations between task performance and apathy, and tested whether both memory and motivational deficits related to hippocampal structure and seed-based functional connectivity.

## Materials and Methods

### Participants

Twenty-seven individuals with a previously established diagnosis of anti-LGI1 aLE (age: *μ* = 65.00 years, s.d. = 9.45 years; 21 males) were tested along with 27 healthy age- and gender-matched controls (age: *μ* = 64.19 years, s.d. = 8.52 years; 21 males). All participants gave written consent to take part in the study and were offered monetary compensation for their time. The study was approved by the University of Oxford ethics committee (RAS ID No. 248,379, Ethics Approval Reference No. 18/SC/0448). Table 1 shows the demographics of the two groups.

**Table 1.** Group comparisons between HC and aLE

Variable	HC (*n* = 27)	aLE (*n* = 27)	*p*-Value	Effect size
Gender (F/M)	6/21	6/21	–	–
Age (years)	64.19 (±8.52)	65.00 (±9.45)	0.741	*d* = −0.09
ACE III total	97.19 (±2.94)	92.04 (±7.08)	0.001	*d* = 0.95
Attention	17.70 (±0.82)	17.41 (±1.34)	0.332	*d* = 0.27
Memory	24.85 (±1.63)	23.22 (±3.03)	0.018	*d* = 0.67
Fluency	12.93 (±1.38)	10.52 (±2.65)	<0.001	*d* = 1.14
Language	25.85 (±0.46)	25.37 (±1.52)	0.126	*d* = 0.43
Visuospatial	15.85 (±0.36)	15.52 (±0.94)	0.093	*d* = 0.47
Digit span total	17.48 (±4.84)	20.11 (±4.99)	0.055	*d* = −0.54
Apathy (AMI)	1.25 (±0.34)	1.33 (±0.57)	0.548	*d* = −0.17
Depression (BDI-II)	5.33 (±4.04)	10.70 (±8.70)	0.006	*d* = −0.79
Anxiety (HADS)	4.48 (±2.26)	5.80 (±3.80)	0.236	*d* = −0.45
Time since event (yrs)	NA	3.08 (±2.74)	NA	NA

Values are Mean (±SD) unless otherwise stated. Time since event (yrs) is the interval between behavioral testing and the last relapse, or diagnosis if no relapse was recorded.

### Clinical measures

Participants completed a battery of validated self-report questionnaires assessing mood, motivation, and cognitive function. Apathy was measured using the Apathy Motivation Index (AMI; Ang et al., 2017), an 18-item questionnaire that assesses diminished motivation across behavioral, social, and emotional domains. Higher scores indicate greater levels of apathy. Depressive symptoms were assessed using the Beck Depression Inventory-II (BDI-II; Beck et al., 1961, 1996), a 21-item self-report measure widely used in clinical and research settings. Anxiety was measured using the anxiety subscale of the Hospital Anxiety and Depression Scale (HADS; Zigmond and Snaith, 1983), a 7-item measure designed for use in medical populations. Global cognitive function was assessed using the Addenbrooke’s Cognitive Examination (ACE III; Bruno and Vignaga, 2019), a clinical screening tool that evaluates attention, memory, verbal fluency, language, and visuospatial abilities, with a maximum score of 100.

### Experimental paradigm and procedure

**Figure 1.**
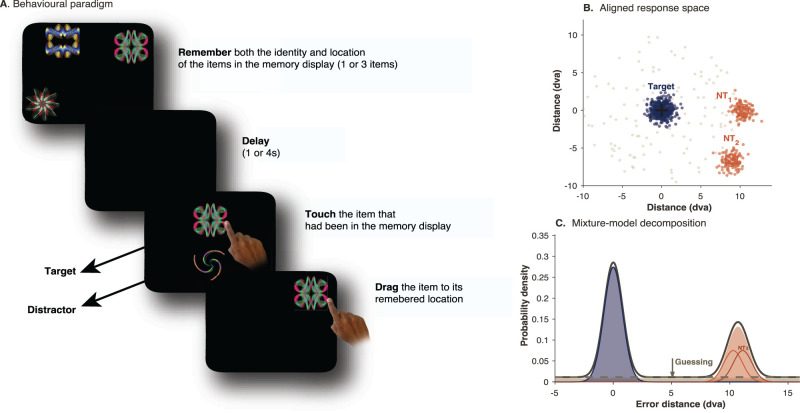
What was where Oxford memory task (OMT). ***A***, Participants were presented with either 1 or 3 items. They were to remember what they looked like and where they were on the screen. After a variable delay (1 s or 4 s), a dual choice was presented. Participants had to touch the one they remember from a few seconds before, then drag it to where it originally appeared. ***B***, Aligned response space (simulation). To aggregate localization errors across trials, each response was re-centered on the target origin and rotated so that the nearest non-target fell along the positive horizontal axis. Target-directed responses (navy) cluster tightly around the origin; misbinding errors (terracotta) cluster around the non-target locations; and guessing responses (sand) are distributed uniformly. ***C***, Mixture-model decomposition of localization error. The distribution of recall errors (black curve) was modeled as a mixture of three components: (1) **Precision (navy):** A von Mises distribution centered on the target location; (2) **Misbinding (terracotta):** A sum of Gaussian distributions centered on each non-target location (the solid filled area shows the aggregate misbinding probability); and (3) **Guessing (sand):** A uniform probability distribution extending across the search space, visualized as a hatched band. This decomposition dissociates distinct sources of working memory failure—degraded precision, feature–location binding errors, and representational loss—within the same behavioral measure.

Participants performed a variant of the “What was where” Oxford memory task (OMT; Pertzov et al., 2013). A schematic of the task is shown in Figure 1. On each trial, participants encoded the identity and location of a briefly presented set of items and, following a delay, selected the previously seen item from a two-alternative display and repositioned it to its original location.

Stimuli were presented on a black background and were drawn from a library of 196 shapes. For each trial, shapes were selected at random without repetition. Stimuli were viewed from approximately 30 cm and subtended a visual angle of approximately 2.3°. Participants sat in front of an interactive touch-sensitive screen [iPad; iOS 9.3.5 (13G36), model MGTX2B/A; 1,536 × 2,048 pixels].

Two factors were manipulated. First, memory load was varied by presenting either one or three items (set size; Fig. 1). Participants were instructed to remember what the items looked like and where they appeared on the screen. The items then disappeared and a blank screen was shown for either 1 or 4 s (delay). After the delay, two shapes were presented: a target (an item from the preceding display) and a distractor (a foil drawn from the same library but not shown in that trial’s memory array). Participants first selected the remembered item by touching it and then dragged it to the location at which it had originally appeared. They were instructed to respond as accurately as possible and were allowed to adjust the item’s position as many times as needed before finalizing their response.

The task comprised four experimental conditions (set size × delay), presented in random order. Conditions were administered across three blocks, each containing 10 trials per condition (40 trials per block), yielding a total of 30 trials per condition (120 trials overall). The number of trials per condition was determined in pilot work to maximize statistical power while keeping the task as brief as possible.

### Behavioral analysis and modeling

The task enabled us to quantify the fidelity of working memory representations by modeling the distribution of recall errors (i.e., recall precision). This approach provides information about the quality of stored representations and allows the underlying sources of error that shape performance to be separated (Bays and Husain, 2008; Bays et al., 2009; Gorgoraptis et al., 2011; Ma et al., 2014).

We defined **identification accuracy** as the proportion of trials on which participants selected the target shape (rather than the foil) in the two-alternative choice. **Identification time** was the latency to make this choice. **Localization error** was indexed by the Euclidean distance between the center of the true target location and the center of the reported location. Localization error was computed only for trials on which the target was correctly identified, thereby isolating spatial fidelity conditional on correct item selection.

To characterize distinct sources of localization error (imprecision, misbinding, and guessing) we fitted an extension to MemToolbox using the 2D mixture-model framework described by Grogan et al. (2020). In this model, **imprecision** reflects the dispersion of responses around the intended (or mistakenly selected) location; **misbinding** captures the proportion of trials on which the correctly identified item is placed at the location of a different item from the memory array; and **guessing** corresponds to uniformly random responses across the display. We additionally derived misbinding over delay as the difference in misbinding probability following a 4-s versus a 1-s retention interval.

### Magnetic resonance data acquisition

Across participants, MR images were acquired on average two months after the behavioral assessment (SD = 3.53) using a 3 T Siemens Verio scanner at the Acute Vascular Imaging Centre, John Radcliffe Hospital, Oxford, UK. The imaging protocol was aligned with the UK Biobank acquisition pipeline; further details are reported elsewhere (Alfaro-Almagro et al., 2018). T1-weighted images were acquired using an MPRAGE sequence with 1 mm^3^ isotropic resolution. Resting-state functional MRI (rs-fMRI) data were acquired at 2.4 mm isotropic resolution using a gradient-echo echo-planar imaging (GE-EPI) sequence (multiband acceleration factor =  8; field of view = 88 × 88; matrix = 88 × 88 × 64; TR/TE = 735/39 ms; flip angle = 52°; fat saturation; no iPAT). T2-weighted fluid-attenuated inversion recovery images were also acquired (192 sagittal slices; slice thickness = 1.05 mm; voxel size = 1 × 1 × 1.1 mm; TR/TE =  5,000/397 ms; readout FOV = 256; iPAT = 2; partial Fourier = 7/8; fat saturation; prescan normalize).

rs-fMRI measures spontaneous fluctuations in blood oxygenation (BOLD signal) attributable to intrinsic brain activity. The rs-fMRI images were a voxel size of 2.4 × 2.4 × 2.4 mm (GE-EPI with multiband acceleration factor = 8, field of view: 88 × 88 × 64 matrix, TR/TE = 735/39 ms, flip angle = 52°, fat saturation, no iPAT).

MR images were obtained from all MRI-compatible participants who consented to be scanned for the study. This included 27 HC and 12 aLE patients.

### Magnetic resonance data analyses

Whole hippocampal and hippocampal subfield volumes were extracted from T1- and T2-weighted images using FreeSurfer v7.1 (http://surfer.nmr.mgh.harvard.edu/) with the standard automated segmentation protocol. Hippocampal volumes were adjusted for intracranial volume (ICV) using the following equation (Jack et al., 1998; Voevodskaya, 2014):

$$V_{\text{adj}}=V-\beta \times (\text{ICV}-\bar{\text{ICV}}),\;(1)$$

where *V*_adj_ and *V* are the adjusted and observed volumes respectively, *β* is the slope of the relationship between ICV and *V* in HC, and $\bar{\text{ICV}}$ is the mean ICV in this control sample.

The CONN toolbox v20.b (Whitfield-Gabrieli and Nieto-Castanon, 2012), running with SPM12, was used to analyze resting-state connectivity in MATLAB R2024b, implementing the default preprocessing pipeline (see *Supplementary information*, Text S3, for details).

Seed-based functional connectivity analyses used the default hippocampal masks generated by CONN during preprocessing. Between-group connectivity differences were tested while controlling for age and gender. Statistical inference used threshold-free cluster enhancement (TFCE), with a cluster-wise corrected significance threshold of *p* < 0.05 based on 5,000 permutations. A control analysis, using amygdala masks (right and left) as seeds was performed to ensure specificity of the results to the hippocampus.

### Statistical analyses

All statistical analyses were performed using MATLAB (The MathWorks inc., version 2024b) with the Statistics and Machine Learning Toolbox. The significance threshold was set at *α* = 0.05 for all analyses unless otherwise specified.

Group differences in demographic and clinical variables were examined using Welch’s *t*-tests for continuous variables, which do not assume equal variances between groups. Gender distributions were compared using Pearson’s chi-square test. Effect sizes were calculated using Cohen’s *d* for continuous variables and the phi coefficient (*ϕ*) for categorical variables.

Memory performance on OMT was analyzed using linear mixed-effects models using *fitlme* function in MATLAB. A 2 × 2 × 2 mixed factorial design was employed with Group (aLE vs Controls) as a between-subjects factor and Delay (1 s vs 4 s) and Set Size (1 item vs 3 items) as within-subjects factors. Models were fitted using restricted maximum likelihood estimation with subject included as a random intercept to account for within-subject correlations. Separate models were fitted for each behavioral outcome: identification time, localization error, and accuracy.

Parameters from the MCMC mixture-model (misbinding probability, guessing probability, and memory precision) were analyzed using linear mixed-effects models with Delay (1 s vs 4 s) as a within-subjects factor and Group as a between-subjects factor. Additional models (between and within groups) examined interactions between task parameters and clinical measures (apathy and depression) to investigate individual differences in memory performance. All models were controlled for age, gender and global cognition (ACE III) scores. When apathy was investigated, the models were also controlled for depression.

Group differences in hippocampal subfield volumes were examined using Welch’s *t*-tests.

Relationships between hippocampal–frontal functional connectivity and both apathy and misbinding were examined using linear models in each group separately.

To identify the most parsimonious model explaining the relationship between apathy, depression, and misbinding, a series of nested linear regression models were compared using information criteria. Model fit was assessed using the Akaike information criterion (AIC; Akaike, 1974) and the Bayesian information criterion (BIC; Schwarz, 1978). Models with lower AIC and BIC values were considered to provide a better balance between model fit and complexity. The models tested ranged from a baseline model including only group membership to a full model including linear and quadratic effects of apathy, depression, and their interaction.

Multiple-comparison correction using the Benjamini–Hochberg false discovery rate (FDR) procedure (*q* = 0.05) was applied to families of secondary or exploratory tests, including hippocampal sub field volume comparisons and sub field-behavior correlations. Primary tests, including the group difference in misbinding errors and the apathy–misbinding association within patients, are pre-specified from hypotheses motivated in the Introduction and are reported without family-wise correction.

## Results

### Demographics

Demographic and clinical characteristics of the study sample are presented in Table 1. The groups were well-matched for age and gender distribution, with no significant differences observed between HC and aLE patients.

Patients with aLE demonstrated significantly lower global cognitive function, as measured by the ACE III, compared to controls (*t* = 3.49, *p* = 0.001, *d* = 0.95). Memory and fluency were the only two ACE III subdomains significantly affected (memory: *p* = 0.018, *d* = 0.67; fluency: *p* < 0.001, *d* = 1.14), while no significant differences were observed in attention, language, or visuospatial domains (all *p* > 0.05). Additionally, aLE patients reported significantly higher levels of depressive symptoms on the BDI-II (*t* = 2.91, *p* = 0.006, *d* = 0.79). Despite these differences, the two groups did not differ significantly in levels of apathy (*p* = 0.548) or anxiety (*p* = 0.236).

### Working memory performance

**Figure 2.**
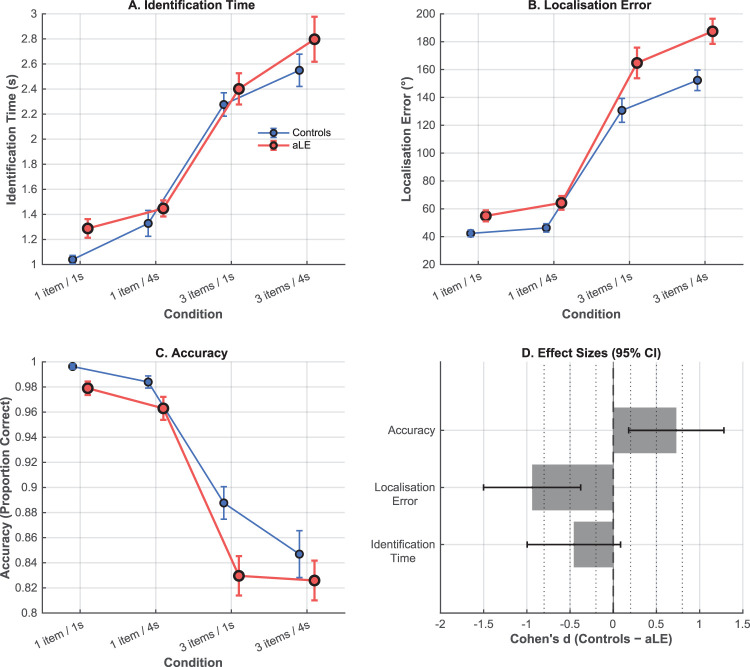
Memory performance. ***A–C***, aLE patients showed worse localization performance (higher error rates) and lower accuracy, compared to healthy matched controls. No difference in identification time was observed between the two groups. Effect sizes of these differences are shown in ***D***, Error bars in ***A–C*** show SEM, and 95% CI in ***D***.

Patients with aLE demonstrated impaired memory performance relative to HC on key measures, alongside robust effects of task difficulty. In the full mixed-effects model including Group, Delay, and Set Size (and all interactions), aLE patients demonstrated higher localization errors [*F*(1, 208) = 12.35, *p* = 5.41 × 10^−4^, Fig. 2*B*], and lower accuracy [*F*(1, 208) = 7.47, *p* = 0.0068, Fig. 2*C*]. By contrast, there was no evidence of a group difference in identification time [*F*(1, 208) = 2.92, *p* = 0.089, Fig. 2*A*], indicating that the observed deficits were not explained by general slowing.

Across groups, performance varied systematically with task demands. Delay and Set Size showed significant main effects on all outcomes [identification time: Delay *F*(1, 208) = 21.21, *p* = 7.18 × 10^−6^; Set Size *F*(1, 208) = 411.66, *p* = 3.35 × 10^−51^; localization error: Delay *F*(1, 208) = 14.72, *p* = 1.65 × 10^−4^; Set Size *F*(1, 208) = 811.40, *p* = 1.00 × 10^−73^; accuracy: Delay *F*(1, 208) = 6.12, *p* = 0.014; Set Size *F*(1, 208) = 326.75, *p* = 1.58 × 10^−44^], consistent with poorer performance at longer delays and higher memory load. Crucially, there was no evidence that group differences were modulated by delay for any measure (all Group × Delay and three-way interactions *p* > 0.25). However, for localization error there was a significant Group × Set Size interaction [*F*(1, 208) = 6.65, *p* = 0.0106], indicating that the magnitude of the group difference varied with memory load. Localization error also showed a Delay × Set Size interaction [*F*(1, 208) = 4.27, *p* = 0.040], whereas no other interaction terms were significant.

Controlling for age, gender, and global cognition (ACE III) yielded a broadly similar pattern to the unadjusted model. There remained no group difference in identification time [*F*(1, 205) = 0.86, *p* = 0.354, Table S2A], and the main effects of Delay and Set Size were unchanged. Localization error continued to show a robust group effect [*F*(1, 205) = 7.85, *p* = 0.0056] alongside a significant Group × Set Size interaction [*F*(1, 205) = 6.65, *p* = 0.0106]. For accuracy, the group effect was attenuated and no longer significant [*F*(1, 205) = 2.85, *p* = 0.093], while ACE III emerged as a significant predictor [*F*(1, 205) = 5.82, *p* = 0.0168], suggesting that between-group differences in global cognitive status contribute to accuracy differences.

### Predominant impairment in working memory binding in aLE

To examine whether patients with aLE exhibited deficits in working memory binding, we fitted generalized linear mixed-effects models with Group (aLE vs controls), Delay (1 s vs 4 s), and their interaction as fixed effects, controlling for age, gender, and global cognition (ACE III). Participant was included as a random intercept. Separate models were estimated for each mixture-model parameter: misbinding, guessing, and precision (Table S2B).

**Figure 3.**
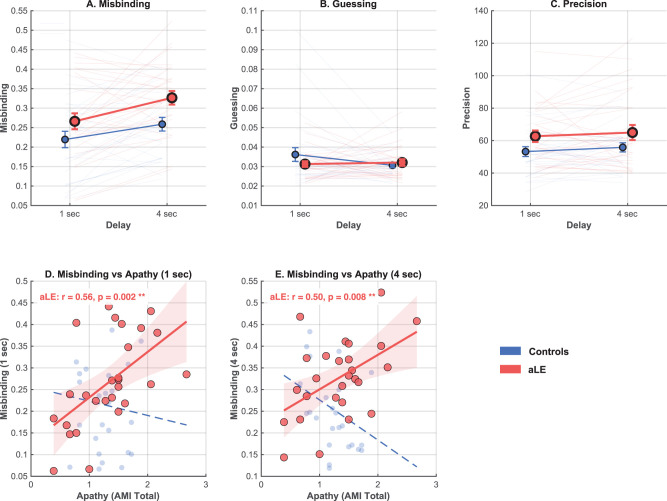
Mixture-model results and relationship with apathy. ***A***, Across delays, aLE patients showed higher misbinding rates compared to healthy controls. ***B, C***, No significant difference was observed between the two groups in guessing and precision. ***D, E***, Apathy was associated with higher misbinding in aLE. Error bars show SEM.

For misbinding, patients with aLE showed significantly higher misbinding rates than controls [*F*(1, 99) = 5.12, *p* = 0.0259; Fig. 3*A*]. Misbinding also varied with retention interval [*F*(1, 99) = 14.28, *p* = 2.69 × 10^−4^], whereas the Group × Delay interaction was not significant [*F*(1, 99) = 0.75, *p* = 0.389], indicating that the group difference was comparable across delays. None of the covariates (age, gender, and ACE III) were associated with misbinding (all *p* > 0.45). The independent contributions of apathy and depression to misbinding are further dissociated in the *Supplementary information* (Text S1; Fig. S1).

For precision, there was a trend toward a group difference that did not reach conventional significance after adjustment [*F*(1, 99) = 3.67, *p* = 0.058; Fig. 3*C*]. Age was significantly associated with precision [*F*(1, 99) = 5.49, *p* = 0.021], while neither Delay nor the Group × Delay interaction was significant (both *p* ≥ 0.29).

For guessing, there was no evidence of a group effect [*F*(1, 99) = 1.30, *p* = 0.257; Fig. 3*B*], nor effects of Delay or Group × Delay (both *p* ≥ 0.11).

Collectively, these findings indicate that aLE is associated with a predominant impairment in memory binding that persists after accounting for age, gender, and global cognitive function, with no evidence for an accompanying increase in guessing and only limited evidence for reduced precision.

### Apathy is associated with misbinding errors in aLE

Having established group differences in misbinding, we next examined whether apathy was associated with these binding deficits. We fitted a model including Group, Delay, Apathy, and their interactions, with age, gender, ACE III, and depression as covariates (Table S2C).

The analysis revealed a significant Group × Apathy interaction [*F*(1, 94) = 6.33, *p* = 0.014], indicating that the relationship between apathy and misbinding differed between groups (Fig. 3*D*,*E*). To decompose this interaction, we examined the apathy–misbinding association within each group separately.

Within the aLE group, greater apathy was significantly associated with increased misbinding errors [*F*(1, 46) = 6.54, *p* = 0.014; Fig. 3*D*,*E*, red], such that patients reporting higher levels of apathy exhibited more frequent binding failures. In contrast, within the control group, apathy showed a significant association in the opposite direction [*F*(1, 44) = 4.54, *p* = 0.039; Fig. 3*D*,*E*, blue], with higher apathy associated with fewer misbinding errors.

The main effect of delay remained significant in the full model [*F*(1, 94) = 14.21, *p* < 0.001], and was also significant within the aLE group [*F*(1, 46) = 11.42, *p* = 0.001], with a trend in controls [*F*(1, 44) = 3.82, *p* = 0.057]. The Delay × Apathy interaction was not significant in the full model [*F*(1, 94) = 1.77, *p* = 0.187], suggesting that the apathy–misbinding relationship did not vary as a function of retention interval; the three-way Group × Delay × Apathy interaction was also non-significant [*F*(1, 94) = 0.29, *p* = 0.592]. None of the covariates—age, gender, ACE III, or depression—were significantly associated with misbinding errors (all *p*s ≥0.35).

These results demonstrate that apathy is specifically linked to binding deficits in patients with aLE, independent of global cognitive impairment and depressive symptomatology, highlighting a potential motivational contribution to memory dysfunction in this population.

### Smaller hippocampal volumes in aLE

We first examined group differences in overall hippocampal volume, given that medial temporal lobe involvement was a key feature of the aLE cohort. Consistent with this, aLE patients showed significantly reduced right whole hippocampal volume relative to controls (*t* = 2.26, *p* = 0.043, *d* = 1.09; Table S3). There was no corresponding difference for the left whole hippocampus (*p* = 0.319), indicating that volumetric reductions were more pronounced on the right in this sample.

To clarify whether this whole-structure difference reflected selective vulnerability of particular hippocampal components, we then conducted follow-up comparisons of hippocampal subfield volumes (subfields constituting the whole hippocampus). At the uncorrected level, the largest group differences were observed in the right presubiculum (*t* = 2.69, *p* = 0.015, *d* = 1.03) and right molecular layer (*t* = 2.23, *p* = 0.045, *d* = 1.04), with a similar trend in the right subiculum (*t* = 2.14, *p* = 0.051, *d* = 0.94). No other subfields showed evidence of group differences, and left-sided subfields were largely comparable between groups (all *p* ≥ 0.17). However, none of the subfield comparisons survived FDR correction (all *q* ≥ 0.254), suggesting that while the pattern of effects was broadly consistent with right-lateralized hippocampal involvement, the available sample did not provide sufficient evidence to localize the whole hippocampus difference to a specific subfield after correction for multiple comparisons.

### Reduced hippocampal–frontal functional connectivity in aLE

**Figure 4.**
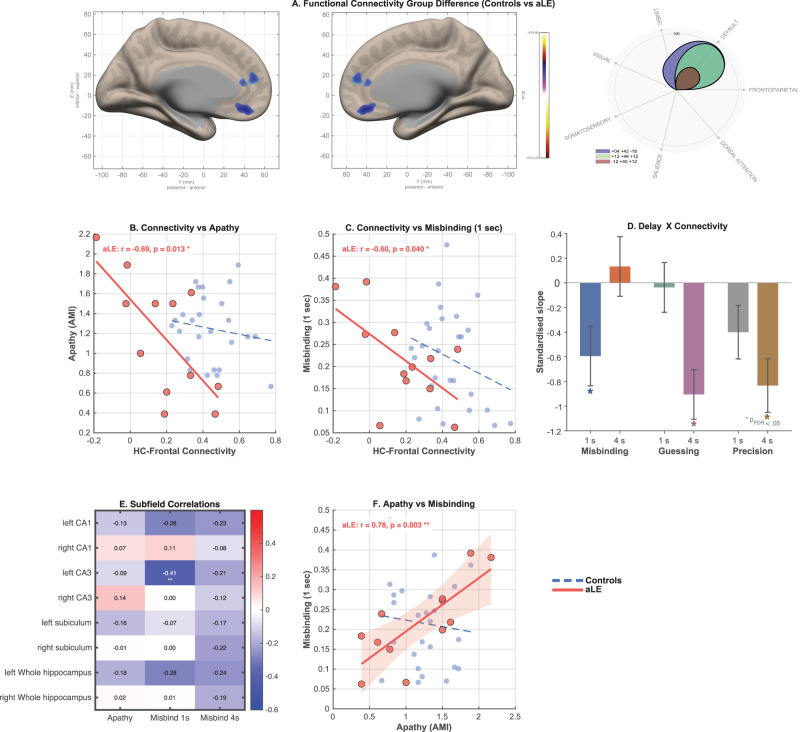
Hippocampal connectivity, misbinding and apathy. ***A***, aLE patients demonstrated reduced resting hippocampal (left) connectivity with medial frontal regions, compared to healthy controls. This connectivity pattern is mapped to limbic, default and frontotemporal networks as shown on the top right panel. ***B–D***, Reduced hippocampal–frontal connectivity was associated with apathy and misbinding, mainly at short intervals (see *Supplementary information* for detailed analysis of delay effects). ***E***, Misbinding errors also correlated negatively with left CA3 subfield volumes. ***F***, The relationship between apathy and misbinding remains significant, for the smaller sample of patients who have MRI data.

We next examined resting-state functional connectivity between the hippocampus and cortical regions using seed-to-voxel analysis. Left and right whole hippocampal seeds were tested separately. Significant group differences emerged for the left hippocampus, where healthy controls exhibited greater connectivity than aLE patients to three frontal clusters (TFCE; all *p*_FWE_ < 0.05; Fig. 4*A*).

The largest cluster (137 voxels; peak: +4, + 42, − 18; TFCE =1074.20, *p*_FWE_ = 0.033) was located predominantly within the medial frontal cortex (66%), with extension into the left paracingulate gyrus (21%). A second cluster (97 voxels; peak: +12, + 46, + 12; TFCE =1023.02, *p*_FWE_ = 0.039) was located almost exclusively within the right paracingulate gyrus (96%). A third, smaller cluster (23 voxels; peak: −12, + 40, + 12; TFCE =975.65, *p*_FWE_ = 0.046) encompassed the anterior cingulate cortex (70%) and left paracingulate gyrus (17%).

When the same analyses were performed using the amygdala as seed region, no significant difference between patients and controls was found.

Collectively, these findings suggest that aLE is associated with reduced left hippocampal connectivity to medial prefrontal regions implicated in memory retrieval and cognitive control. No significant differences were observed for right hippocampal connectivity. However, these connectivity analyses were conducted in a subset of 12 aLE patients with usable resting-state data and 27 controls, and therefore should be interpreted as exploratory and hypothesis-generating.

### Associations between connectivity, apathy, and misbinding

To investigate whether reduced left hippocampal–frontal connectivity was related to clinical and behavioral outcomes, we extracted mean connectivity values with the significant medial prefrontal cluster (cluster 1) and examined its correlation with apathy and misbinding (Fig. 4*B*–*D*).

Within the aLE group, left hippocampal–frontal connectivity was strongly negatively correlated with apathy (*ρ* = −0.69, *p* = 0.013, *n* = 12; Fig. 4*B*), such that patients with weaker connectivity reported higher levels of apathy. This association was not significant in controls (*ρ* = −0.15, *p* = 0.47).

Left hippocampal–frontal connectivity was also significantly associated with misbinding errors at the short retention interval in aLE patients (*ρ* = −0.60, *p* = 0.040; Fig. 4*C*), with weaker connectivity predicting more binding failures. This relationship was not observed at the longer retention interval (*ρ* = 0.15, *p* = 0.64; Fig. 4*D*) or in controls (both *p* > 0.17). To formally test the delay-specificity of this association, we fitted linear mixed-effects models with Delay (1, 4 s), centered connectivity, and their interaction as fixed effects and subject as a random intercept. The Delay × Connectivity interaction was significant (*χ*^2^(1) = 10.45, *p* = 0.001) and was unchanged by inclusion of apathy as a covariate (*β* = 0.374 in both models). Complementary analyses of guessing and precision errors revealed the opposite delay-specificity, with connectivity predicting both error types selectively at 4 s (all FDR-corrected; Fig. 4*D*; see *Supplementary information*, Text S2, Table S1 and Fig. S2).

Consistent with our earlier findings, apathy remained significantly correlated with misbinding in the imaging subset of aLE patients at the 1-s delay (*ρ* = 0.75, *p* = 0.005) and when averaged across delays (*ρ* = 0.68, *p* = 0.014; Fig. 4*F*), confirming that the apathy–misbinding relationship is robust even in this smaller sample with neuroimaging data.

Finally, correlations between hippocampal subfield volumes and behavioral measures controlled for age and gender revealed an association between CA3 volume and misbinding at the 1-s delay across all participants (*r* = 0.41, *p* = 0.0056; Fig. 4*E*). However, this did not survive an extensive FDR correction threshold across 24 multiple comparisons (*p*_FDR_ = 0.09).

In summary, patients with aLE demonstrated reduced left hippocampal functional connectivity to medial prefrontal and anterior cingulate regions. Within the patient group, weaker hippocampal connectivity with medial prefrontal region was associated with both higher apathy and increased misbinding errors at short delays. These findings suggest that disrupted left hippocampal–prefrontal communication may contribute to both the motivational and mnemonic deficits observed in aLE, potentially through a common neural mechanism linking memory binding failures to apathy.

## Discussion

This study shows that LGI1-antibody encephalitis (aLE), a disorder known to produce focal hippocampal atrophy (Miller et al., 2017; Spanò et al., 2020a,b; Attaallah et al., 2024b), is associated with predominant impairment in working memory binding, reduced hippocampal–prefrontal connectivity, and a novel link between misbinding, apathy and hippocampal–frontal connectivity. These findings broaden current accounts of hippocampal function beyond its conventional association with long-term memory, indicating that the hippocampus also contributes to short-term memory processes and to aspects of motivated behavior, likely through partially overlapping neural mechanisms.

Patients with aLE showed significantly elevated misbinding errors, with comparable guessing rates and only marginally reduced precision, indicating a predominant deficit in binding object identities to spatial locations rather than generalized memory degradation. This aligns with previous reports showing that the hippocampus supports high-resolution binding regardless of retention interval (Olson et al., 2006; Finke et al., 2008; Pertzov et al., 2013; Watson et al., 2013; Yonelinas, 2013; Liang et al., 2016), and extends earlier work by Pertzov et al. (2013) who used the same “What was where?” task in aLE. Subsequent studies reported object–location misbinding deficits in AD spectrum, with hippocampal volume negatively correlating with swap error rates (Liang et al., 2016; Toniolo et al., 2025a). The present study extends these observations by demonstrating that binding deficits in aLE correlate with motivational dysfunction and are accompanied by alterations in hippocampal functional connectivity.

A key novel finding of this study is the robust positive association between apathy and misbinding errors in aLE patients. This relationship remained significant after adjusting for depressive symptoms and global cognitive status, which are also common with aLE (van Sonderen et al., 2016; Binks et al., 2021; Kelly et al., 2025). This indicates that apathy is linked to binding deficits via mechanisms that are at least partly dissociable from general cognitive decline or mood disturbance. In contrast, the opposite pattern in HC—where higher apathy scores were associated with fewer misbinding errors—suggests that the apathy—misbinding association is pathologically specific to the patient group rather than reflecting a generic trait effect.

One possible explanation of this opposite pattern between patients and controls is that apathy does not index the same underlying process across groups. In patients, apathy is more plausibly a disease-related symptom linked to disruption of hippocampal–prefrontal and broader motivational circuitry, making its association with impaired binding mechanistically interpretable. In HC, by contrast, apathy scores are range-restricted, which makes correlations more vulnerable to instability and limits direct comparison with patients. Alternatively, if the control association is robust, apathy in patients might partly reflect a compensatory response to memory impairment, whereby withdrawal from effortful goal pursuit preserves performance. The present cross-sectional data cannot distinguish between these possibilities or determine temporal ordering. The absence of group differences in apathy scores and the lack of structural correlates may also reflect limited sensitivity of self-report apathy measures relative to caregiver reports (Klar et al., 2022; Attaallah et al., 2024a), and of gross hippocampal volume relative to functional connectivity measures.

From a mechanistic perspective, apathy has traditionally been linked to deficits in goal-directed behavior and decision-making, particularly in the weighing of costs and benefits when selecting and executing actions (Husain and Roiser, 2018; Le Heron et al., 2019). Recent theoretical work has expanded this view, proposing that apathy reflects impairments in active inference and executive control processes (Williams and Rowe, 2025). In AD, for example, apathy severity correlates with the degree of executive dysfunction and with reduced hippocampal–frontal connectivity (Attaallah et al., 2024a), consistent with impaired integration of mnemonic context into ongoing goal-directed behavior. Conversely, impulsivity, at the opposite end of the motivational spectrum, has been linked to failures in evidence accumulation that also implicate hippocampal networks supporting prospective cognition and uncertainty processing (Wimmer and Shohamy, 2012; Shohamy and Daw, 2015; Attaallah et al., 2024b, 2025).

Under this view, goal-directed behavior requires mental simulation of future outcomes via hippocampal-dependent recombination of past experience (Gilbert and Wilson, 2007; Schacter et al., 2007; Buckner, 2010; Addis et al., 2011; Addis and Schacter, 2012), a computational operation analogous to working memory binding. Our finding that binding failures correlate with apathy in patients with hippocampal damage is consistent with the hypothesis that a common relational processing mechanism may underlie both memory binding and aspects of motivated, future-oriented behavior.

An important unresolved question is whether apathy arises as a consequence of degraded memory and executive processes, with individuals losing motivation due to dysfunctional contextual representations of goals and their associated rewards, or whether apathy represents a distinct behavioral construct that secondarily impairs memory by reducing the salience of items deemed unworthy of encoding. The latter possibility raises intriguing questions about whether motivational states modulate binding even for stimuli without intrinsic reward value. Regardless of the precise causal architecture, the co-occurrence of apathy and memory binding deficits carries important clinical implications. Apathy is recognized as an early marker of dementia and is prevalent across many neurodegenerative disorders, often emerging years prior to clinical diagnosis (Van Dalen et al., 2018; Lanctot and Aleman, 2021). Pharmacological interventions that enhance cholinergic function have been shown to improve both cognitive performance and apathetic symptoms (Emre et al., 2004; Devos et al., 2014), suggesting that these domains may share common neurochemical substrates amenable to therapeutic intervention.

Structural imaging revealed reduced right whole hippocampal volume in patients with aLE, consistent with previous neuroimaging reports showing focal hippocampal damage in this patient group (Finke et al., 2017; Miller et al., 2017; Spanò et al., 2020a,b; Attaallah et al., 2024b). At the subfield level, the largest effect sizes were observed in the right presubiculum and molecular layer, although these did not survive correction for multiple comparisons. The modest sample size for neuroimaging analyses (12 patients) limits the ability to detect focal subfield effects. Resting-state functional connectivity analyses revealed that patients with aLE showed reduced connectivity between the left hippocampus and medial prefrontal regions, including the medial frontal cortex, paracingulate gyrus, and anterior cingulate cortex. These regions constitute key nodes of the default mode network and have been implicated in self-referential processing, memory retrieval, and goal-directed behavior (Buckner et al., 2008; Andrews-Hanna et al., 2010). Disrupted hippocampal–prefrontal connectivity has been reported in AD (Wang et al., 2006; Allen et al., 2007), Parkinson’s disease (Skidmore et al., 2013), and healthy ageing (Salami et al., 2014), and has been linked to both memory impairment and motivational deficits across these conditions.

Intracranial recordings have begun to clarify how hippocampal–frontal interactions support working memory. Daume et al. (2024) showed that hippocampal theta–gamma phase–amplitude coupling tracks both memory load and quality over short durations, and that hippocampal neurons become coordinated with frontal theta oscillations when cognitive control demands are high. Such findings suggest that the reduced hippocampal–frontal connectivity observed in aLE may impair the coordinated oscillatory dynamics required for successful working memory binding.

The left-lateralized connectivity differences, occurring alongside right-lateralized atrophy, may reflect either a maladaptive compensatory response in the less-affected hemisphere or a more domain-general role of left hippocampus in relational processing, since the task here was visuospatial rather than verbal. The asymmetry should be treated as preliminary given the small imaging subsample and requires replication.

These findings have several implications for the clinical management of patients with aLE. First, persistent binding deficits in the chronic phase highlight the need for cognitive rehabilitation strategies targeting working memory; standard tests such as ACE III may underestimate these residual symptoms (Finke et al., 2017), and sensitive binding measures could serve as endpoints for therapeutic interventions. Second, the apathy–misbinding link suggests that motivational symptoms in aLE are not entirely independent of cognitive dysfunction, and interventions targeting hippocampal–prefrontal connectivity may yield benefits for both memory and motivation. Third, our findings underscore the importance of comprehensive neuropsychiatric assessment: apathy is often underrecognized relative to seizures and amnesia despite its substantial impact on quality of life, supporting systematic screening using validated instruments at follow-up.

Some limitations apply. The cross-sectional design restricts causal conclusions about the relationship between connectivity, binding, and apathy; longitudinal studies tracking these measures from disease onset through recovery would help clarify their temporal relationships. The neuroimaging subsample was relatively small (12 patients), limiting statistical power to detect subtle effects and to perform additional analyses such as mediation; effect sizes should therefore be regarded as preliminary, and replication in larger cohorts using higher-resolution MRI is warranted. Resting-state connectivity provides only indirect evidence of functional interactions; task-based connectivity during memory encoding and retrieval would offer more direct insight into the neural dynamics underlying short-term processes.

Overall, the present study demonstrates that patients with hippocampal dysfunction secondary to LGI1-antibody encephalitis exhibit predominant deficits in working memory binding, accompanied by reduced hippocampal–prefrontal connectivity. Critically, binding impairments correlate with apathy, suggesting that the hippocampus supports both working memory and aspects of motivated behavior through partially overlapping mechanisms. These findings advance the emerging view of the hippocampus as a hub that integrates memory, prediction, and goal-directed action, underscoring the need for interventions that target this integrative function in individuals with hippocampal pathology.

## Consent Statement

All participants provided informed consent.

## Data Availability

De-identified data supporting this study may be shared based on reasonable written requests to the corresponding author.

## Code Accessibility

Code for replicating the main results may be shared based on reasonable written requests to the corresponding author.
